# A Comparative Approach of Tumor-Associated Inflammation in Mammary Cancer between Humans and Dogs

**DOI:** 10.1155/2016/4917387

**Published:** 2016-12-08

**Authors:** Maria Isabel Carvalho, Ricardo Silva-Carvalho, Isabel Pires, Justina Prada, Rodolfo Bianchini, Erika Jensen-Jarolim, Felisbina L. Queiroga

**Affiliations:** ^1^CECAV, University of Trás-os-Montes and Alto Douro, 5001-801 Vila Real, Portugal; ^2^Department of Veterinary Sciences, University of Trás-os-Montes and Alto Douro, 5001-801 Vila Real, Portugal; ^3^Centre of Biological Engineering (CEB), University of Minho, Campus de Gualtar, 4710-057 Braga, Portugal; ^4^The Interuniversity Messerli Research Institute, The University of Veterinary Medicine Vienna, Medical University Vienna and University Vienna, Vienna, Austria; ^5^Institute of Pathophysiology and Allergy Research, Center for Pathophysiology, Infectiology and Immunology, Medical University Vienna, Vienna, Austria; ^6^Center for the Study of Animal Sciences, CECA-ICETA, University of Porto, 4051-401 Porto, Portugal; ^7^Center for Research and Technology of Agro-Environment and Biological Sciences (CITAB), University of Trás-os-Montes and Alto Douro, 5001-801 Vila Real, Portugal

## Abstract

Infiltrating cells of the immune system are widely accepted to be generic constituents of tumor microenvironment. It has been well established that the development of mammary cancer, both in humans and in dogs, is associated with alterations in numbers and functions of immune cells at the sites of tumor progression. These tumor infiltrating immune cells seem to exhibit exclusive phenotypic and functional characteristics and mammary cancer cells can take advantage of signaling molecules released by them. Cancer related inflammation has an important role in mammary carcinogenesis, contributing to the acquisition of core hallmark capabilities that allow cancer cells to survive, proliferate, and disseminate. Indeed, recent studies in human breast cancer and in canine mammary tumors have identified a growing list of signaling molecules released by inflammatory cells that serve as effectors of their tumor-promoting actions. These include the COX-2, the tumor EGF, the angiogenic VEGF, other proangiogenic factors, and a large variety of chemokines and cytokines that amplify the inflammatory state. This review describes the intertwined signaling pathways shared by T-lymphocytic/macrophage infiltrates and important tissue biomarkers in both human and dog mammary carcinogenesis.

## 1. General Introduction

Most of the knowledge on tumor biology is derived from human and rodent studies. However, systematic comparison to spontaneous tumors in canine cancer patients could not only contribute to the improved understanding of the disease but contribute also to “translating clinical trials from human to veterinary oncology and back” [1]. This review aimed to compare, in both species, the relevant findings on the role of the immune regulation in mammary cancer.

Tumors are recognized as organs with high complexity and infiltrating immune cells are increasingly accepted to be generic constituents of them [2, 3]. Solid cancers often show signs of inflammation and are infiltrated by many leukocyte populations including T-lymphocytes and macrophages [4, 5]. The role of inflammation in carcinogenesis is not new. Over a century ago, a causal relationship between chronic inflammation and cancer formation was proposed taking into consideration the observations that cancers frequently develop at sites of chronic inflammation [6–8].

Chronic inflammatory responses associated with tumor sites have a multifaceted role in carcinogenesis. Indeed, the chronic inflammation contributes to the acquisition of different hallmark capabilities by incipient neoplasms. Inflammatory cells can induce genomic instability, alterations in epigenetic events, and consequent inappropriate gene expression [9, 10]. Furthermore, immune cells can provide bioactive molecules to the tumor microenvironment, including (i) growth factors that sustain proliferative signaling; (ii) survival factors that limit cell death; (iii) proangiogenic factors and extracellular matrix-modifying enzymes facilitating angiogenesis, invasion, and metastasis; and (iv) inductive signals that lead to activation of other hallmark-facilitating programs [10–13].

During tumor progression, changes in tumor microenvironment induce a switch in innate immune cells toward a protumorigenic function and actively contribute to immune tolerance, preventing rejection of the tumor by the immune system [2, 14]. In this process, dendritic cells (DCs) maturation can be suppressed by changes in the cytokine balance (increased VEGF, TGF*β*, IL-10, IL-6, and COX-2 and reduced IL-4, IL-12, IFN-*α*, and IFN-*γ*) within the tumor, which significantly impairs the antigen presenting function of these cells [8].

Mammary cancer remains a major clinical challenge with considerable mortality both in humans and in dogs [15–18]. Over the years, research on the association between inflammation and mammary cancer pathogenesis has blossomed in both species [19–22]. Alterations of the inflammatory components within the tumor microenvironment have a significant role during carcinogenesis and recent studies have highlighted the importance of tumor-associated immune cells and their influence on neoplastic progression [17, 23–26].

**Figure 1:**
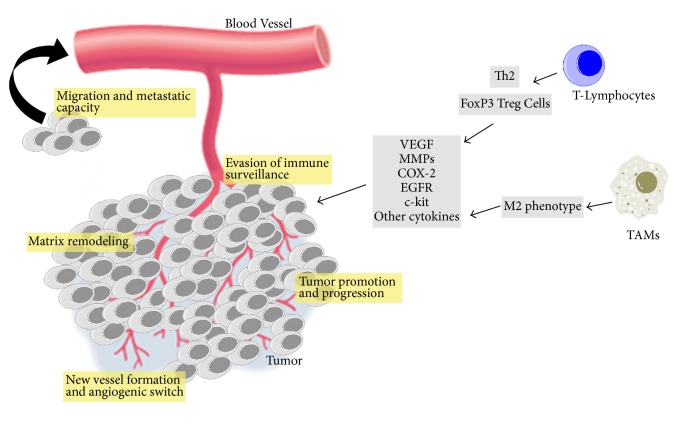
Cancer related inflammation has an important role in mammary carcinogenesis, contributing to tumor evasion of immune surveillance, matrix remodeling and angiogenic switch, acquisition of metastatic capacity, and tumor proliferation and progression.

This review summarizes the intertwined signaling pathways shared by T-lymphocytic/macrophage infiltrates and important tissue biomarkers in both human and dog mammary carcinogenesis (all the contents are synthetized in Table 1 and illustrated in Figure 1). The inflammatory responses in mammary cancer sites are able to orchestrate hallmark-facilitating programs in the tumor microenvironment [27]. These phenomena could be important for prognosis as well as for the development of therapies aimed at redirecting the immune cell actions toward tumor destruction.

## 2. Inflammatory Cells and Sustained Angiogenesis

Similar to normal tissues, vasculature has an important role in tumor growth and progression since it provides the necessary oxygen and nutrients that are present in the blood and allows discarding the toxic waste-products of metabolism [28, 29]. The tumor vasculature, driven by angiogenesis, is thus crucial for growth and survival of tumor cells, but it may also be exploited as a target for cancer therapy [30, 31].

The association between angiogenesis and mammary tumors has been a common subject of interest in humans and dogs [32–34]. In fact, human breast cancer progression is associated with robust angiogenic activity [35]. In highly metastatic human breast cancer there is an upregulation in the expression of proangiogenic factors, such as VEGF [36]. Similarly, in veterinary medicine, the presence of proliferating endothelial cells and blood vessels in intratumoral and peritumoral regions of benign and malignant canine mammary tumors was shown, with the peritumoral regions having the largest blood vessel area and perimeter. Furthermore, malignant tumors have significantly more new vessels and proliferating endothelial cells compared with benign tumors and normal mammary gland tissues [32]. We have shown previously that high microvessel density in canine mammary cancer was significantly correlated with tubule formation, with the histological grade of malignancy and with clinical stage [37].

Infiltrating immune/inflammatory cells secrete a diverse repertoire of proinflammatory mediators, such as cytokines, chemokines, growth factors, and prostaglandins, which trigger the influx of even more inflammatory immune cells to the tumor microenvironment. Furthermore, some of these proinflammatory mediators directly stimulate the migration and proliferation of endothelial cells, thus promoting angiogenesis and consequently tumor growth [10, 38]. T-cells have an important role in the initiation and progression of inflammation by secreting a large number of cytokines and chemokines [39]. It was shown in* in vitro *studies that T-cells in inflammatory sites are in intimate contact with endothelial cells and influence angiogenesis. In fact, T-cells can secrete VEGF by specific antigens stimulation or by IL-2 and by hypoxia [39]. The human invasive breast carcinoma had a higher FoxP3 expression compared to the ductal carcinoma in situ and the adjacent normal tissues. Intratumoral levels of FoxP3, TGF*β*1, and VEGF were found to be positively correlated to each other [40]. TGF*β*1, by T-cell receptor (TCR) stimulation, induces FoxP3 expression in naive CD4^+^CD25^−^FoxP3^−^ T-cells and converts them into FoxP3^+^ regulatory T-cells. Furthermore, Treg cells enhance the TGF*β*1 effects thereby creating a positive autoregulatory loop of TGF*β*1 signaling in CD4^+^CD25^−^ T-cells that potentially stabilizes their regulatory phenotype [41, 42]. The TGF*β*1 and FoxP3 shared pathways induce an upregulated expression of VEGF which increases cancer vascularity and progression [40]. DCs are immunomodulatory cells that initiate adaptive immune responses and exert proangiogenic effects in the tumor microenvironment. Immature DCs promote angiogenesis and tumor growth, whereas mature DCs are known to suppress angiogenesis. Using a mouse model of human breast carcinoma it was observed that rapid tumor growth is associated with the infiltration of immature DCs [43]. Tumor-derived VEGF-A is the main angiogenic factor that prevents DCs maturation by inhibiting the activation of the NF-*κ*B via VEGFR-1 signaling [44]. Furthermore, another study reported that in human breast cancer DCs are differentiated into a phenotype that induces the expansion of Tregs by the expression of IL-10 and TGF*β*1 [45], and the latter is a factor that indirectly induces the expression of VEGF [40]. Monocytes which become tumor-associated macrophages (TAMs) when entering the tumor also act as proangiogenic factor in human breast cancer [46]. In fact, the tumor microenvironment polarizes macrophages toward the M2 phenotype, which is characterized by elevated expression of potent proangiogenic factors. A transgenic mouse susceptible to mammary cancer confirmed that both the angiogenic switch and the progression to malignancy are regulated by infiltrated macrophages in the primary mammary tumors [47]. TAMs are thus responsible for the production of VEGF, of urokinase-type plasminogen activator (uPA), and of matrix metalloproteinase-9 (MMP-9) in human breast carcinomas [46, 48]. Additionally, some studies in human breast cancer showed that the number of macrophages present in tumor sites directly correlates with increased microvessel density, tumor size, and cell proliferation [49, 50]. Specifically, the infiltration of TAMs that express the chemokine CCL18 was positively associated with microvessel density in breast cancer. In fact, the synergistic expression of CCL18 and VEGF by the TAMs promoted endothelial cell migration and angiogenesis in this type of cancer [51].

Some studies from veterinary oncology report that in canine mammary tumors proinflammatory mediators produced by the tumor-infiltrated inflammatory cells correlate with progression and angiogenesis [20, 52–54]. In accordance, we show in dog mammary tumors a positive association between infiltrating CD3^+^ T-cells, VEGF, and microvessel density, implying that CD3^+^ T-lymphocyte cytokines in this type of cancer may stimulate angiogenesis through the induction of the proangiogenic VEGF. Additionally, this high CD3/VEGF expression was associated with an elevated grade of malignancy, presence of neoplastic intravascular emboli, presence of lymph node metastasis, and poor prognosis [52]. It was further shown that myeloid-derived suppressor cells (MDSCs) were significantly increased in stages III and IV dog mammary tumors and in this case MDSCs had significantly altered molecular pathways expressing amplified activation of IL-28/IL-28RA (IFN-*γ*) signaling. Moreover, IL-28 secreted by the MDSCs stimulates the STAT3 pathway which increases VEGF-C expression and therefore induces angiogenesis [55]. A positive correlation between microvessel density and mast cells in malignant canine mammary cancer was found, which, in turn, suggests that these immune cells play an important role in canine mammary cancer angiogenesis, similar to human breast cancer [53]. TAMs in canine mammary cancer have already been associated, by our group, with skin ulceration, histological type, nuclear grade, tubular differentiation, and decrease in overall survival [56]. Similarly to human breast cancer, in canine mammary cancer the macrophages also polarize toward the M2 phenotype [57]. Recently, we and others demonstrated that TAM infiltration is significantly associated with VEGF expression in malignant canine mammary tumors and that genes involved in angiogenic cellular pathways have been significantly upregulated in macrophages cocultured with canine mammary tumor cell lines [20, 58].

## 3. Inflammatory Cells, Tissue Invasion, and Metastasis

Cancer, in its most aggressive form, is a disease characterized not only by uncontrolled cell proliferation and growth but also by uncontrolled cell migration. The activation of angiogenic vasculature is very important for cells to amplify locally (i.e., malignant transformation) and/or spread systemically (i.e., metastasize) [59]. Cancer metastasis involves a complex sequence of processes starting with local invasion, followed by entry of the cancer cells into blood or lymphatic vessels, extravasation in distant tissues, and formation of micrometastasis, followed then by progression into macroscopic tumors [29].

The cross talk between cancer cells and other cells present in the tumor microenvironment is decisive for invasive growth and metastasis. In fact, tumor invasion and metastasis can be potentiated by an inflammatory infiltrate in tumor sites [85]. In addition to promoting angiogenesis, inflammatory infiltrates promote metastatic dissemination by enhancing migratory/invasive potential of neoplastic cells through the production of tissue remodeling proteases, cytokines, and growth factors [86, 87]. Human breast cancer development is characterized by a significant increase in lymphocytes in the neoplastic stroma [60]. For instance, Th2-polarized CD4^+^ T-lymphocytes that express IL-4 promote invasion and subsequent metastasis of mammary adenocarcinomas by regulating the polarization and effector function of TAMs. In turn, M2-TAMs enhance metastasis through activation of EGFR signaling in human malignant mammary epithelial cells [60]. In mice bearing mammary tumors it was demonstrated that IL-1*β* elicits IL-17 expression by gamma delta T-cells, resulting in expansion and polarization of neutrophils by granulocyte colony-stimulating factor (G-CSF) action. Tumor-induced neutrophils acquire the ability to suppress cytotoxic CD8^+^ T-lymphocytes, which favors the metastatic spread [61].

Hence, even the recruitment of monocytes to the tumor has an important role. In fact, CCL2 synthesized by metastatic tumor cells is critical for recruitment of a subpopulation of CCR2 expressing monocytes that enhance the subsequent cell survival and extravasation through VEGF and M-CSF production [88].

Multiple studies in human cancers, including breast cancer, have reported that the presence of TAMs correlates with aggressive disease and outcome [89]. Structural changes in the extracellular matrix are necessary for cell migration. The proteolytic activities of MMP-2 and MMP-9, expressed by macrophages, promote the release of cryptic fragments by cleaving laminin-5 *γ*2 chains which mimic EGF ligand and induce cell motility and invasion in EGFR overexpressing human breast carcinoma cell lines [46, 62]. The coculture with M1 and M2 macrophages increased migration of ER-positive breast cancer cell lines [90]. In a study using a mouse model for breast cancer it was demonstrated that CSF1 may promote metastatic potential by regulating the infiltration and function of TAMs [91]. In fact, using a metastatic breast cancer model it was possible to comprehend that the paracrine loop signaling between TAMs, which supply EGF, and breast cancer cells, which on the other hand supply CSF1, is sufficient for the promotion of invasion and migration [92, 93]. CCL18 released by breast TAMs promoted the invasiveness of cancer cells by triggering integrin clustering and enhancing their adherence to the extracellular matrix [63]. Another study showed that macrophage migration inhibitory factor (MIF) also promoted tumor metastasis by increasing the prevalence of a highly immunosuppressive subpopulation of MDSCs within the tumor [94].

There are few studies in canine mammary cancer regarding the influence of immune cells in tumor invasion and metastasis [55, 64, 95]. The role of lymphocytes and macrophages in canine mammary tumor metastasis is not fully understood. When a high number of CD8^+^ T-cells were found in metastatic canine mammary cancer the authors suggested the involvement of this lymphocyte subtype in tumor metastasis [96]. Contrary to this study, another group showed that the relative percentage of CD4^+^ T-cells was higher in canine mammary tumors that metastasized, whereas CD8^+^ T-cells percentage was higher in tumors that did not metastasize [65, 66]. In terms of TAMs, their density was significantly higher in canine mammary adenocarcinomas that metastasized [64]. Furthermore, a microarray analysis to determine the global gene expression of a coculture of canine macrophages and canine mammary cancer cells showed an upregulation of genes involved in angiogenesis in TAMs and an increase in the migratory and invasive capabilities of cancer cells [95]. Mucha et al. showed, for the first time, that MDSCs demonstrated increased activation of IL-28/IL-28RA (IFN-*γ*) signaling, which stimulates STAT3 in canine mammary tumor cells therefore promoting epithelial-mesenchymal transition (EMT) and increased invasion and migration [55].

## 4. Inflammatory Cells and Cancer Cell Biomarkers

### 4.1. Inflammatory Infiltrates and Tumor COX-2 Expression

Cyclooxygenase (COX) is the enzyme responsible for the biosynthesis of various prostanoids (lipid mediators that have several biological functions) by the conversion of arachidonic acid released from the phospholipid membrane through the action of cytosolic phospholipase A2 [97].

Human and laboratory animal studies report COX-2 upregulation in mammary cancer [98–101] and several lines of evidence now strongly support that this enzyme, during mammary tumorigenesis, mediates tumor survival by several mechanisms: (i) inhibiting the tumor cell apoptosis and inducing tumor cell proliferation; (ii) increasing tumor progression by altering cells morphology; (iii) increasing cell motility and migration; (iv) sustaining proliferative signaling; (v) inducing the production of metastasis-promoting MMPs; and (vi) stimulating the tumor angiogenic switch [102, 103].

The COX-2-derived products, mostly prostaglandin (PG) E2 (thought to be the main tumorigenic COX-2-derived product), are known to act not only in classical cancer signaling pathways to promote carcinogenesis in primary tumor cells but also in the tumor microenvironment which contains multiple resident and infiltrating cells (including immune cells) as well as the growth factors and cytokines released by them [29, 67]. Recent findings revealed that COX-2-produced prostaglandins are potent lipid molecules that act as immunomodulators in key aspects of mammary tumor immunity [68, 104–106].

In human breast cancer COX-2-derived PGE2 has the ability to influence local immune responses in the tumor stroma contributing to tumor evasion of immune surveillance and supporting tumor development and metastasis [102, 106]. PGE2 has diverse effects on the regulation and activity of T-cells [106, 107]. Recently, PGE2 has been implicated in the enhancement of protumorigenic Th2-type cytokine, such as IL-4, IL-5, and IL-10, and inhibition of the anti-tumor Th1 cytokine production, such as IFN-*γ* and IL-2 [67, 68]. Receptors EP1–4, associated with different intracellular signaling pathways, are responsible for mediating the cellular effects of PGE2 [70]. The inhibition of the Th1 cell proliferation is dependent on EP2 [108]. The EP2 and perhaps the EP4 receptors mediate the suppressive effects of PGE2 on T-cells [109]. The induction of the Th2 response by PGE2 is modulated most probably by the second-messenger cyclic adenosine monophosphate (cAMP), since the biological products that increase the level of cAMP mimic the effects of PGE2 [110, 111].

Even though there is limited knowledge regarding the effects of PGE2 on CD8^+^ cytotoxic T-cells, it has been shown that, as for Th1 cells, PGE2 can inhibit CD8^+^ T-cell proliferation, suppress cytotoxic CD8^+^ T-cell actions against the tumor, and, in terms of regulating cytokine production, decrease the production of IFN-*γ* by CD8^+^ T-cell clones through a cAMP-dependent pathway [69]. Additionally, the PGE2 effects during the priming of DCs with tumor antigens inhibit completely the DCs ability to produce IL-12 and prompt the production of high levels of IL-10 [112]. In this process the PGE2-primed DCs induce the direct differentiation of naive T-cells into Th2 cells, which further supports the role of COX-2-derived PGE2 in biasing the immune system toward Th2 and away from potentially beneficial Th1 responses in tumor sites [112, 113].

COX-2 has some effects on FoxP3 expression and Treg cell functions. Several studies have demonstrated that Treg cells contribute to immunosuppression in cancer and inhibit effector T-cells in a COX-2-dependent manner in mouse models or in human peripheral blood [114–117]. An intratumoral increase in COX-2 and PGE2 levels is strongly correlated with the upregulation of FoxP3 and the suppressive capabilities of Tregs in several human cancers [118, 119]. In human breast cancer, COX-2-derived PGE2, acting through EP2 and EP4, increases Treg infiltration, differentiation, and function, which in turn suppress the maturation of other T-cells leading to immunosuppression and increased tumor cell survival [70].

Evidences from clinical and experimental studies indicate that macrophages are versatile cells that are capable of displaying different functional activities in order to promote breast cancer progression and metastasis [20, 120, 121]. TAMs polarization in mammary tumor sites are modulated by the tumor microenvironment and these cells are “educated” adopting a role that facilitates angiogenesis, matrix breakdown, and tumor cell motility in a COX-2-dependent manner [71, 105].

A substantial body of work indicates the immune suppressive role of COX-2 and describes the COX-2/PGE2 modulation of macrophages, including the downregulation of M1 macrophage markers/cytokines, which elicit Th1 immune responses, and the upregulation of M2 macrophage markers/cytokines, which block Th1 immune responses [71, 72]. Moreover, in the murine breast cancer model, the selective COX-2 inhibitors can change the tumor-associated macrophage phenotype from M2 to M1 [105].

Considering COX-2/PGE2-mediated immunomodulation in human breast cancer, it is worth noting that the immunosuppressive cell subtypes, modulated by COX-2 pathways, share common cytokine mediators and, more importantly, each cell subtype can also generate PGE2 providing an autocrine mechanism for prolonging and enhancing their own immunosuppressive phenotype [122]. This emphasizes the importance of exploring the tumor microenvironment as a whole, rather than focusing on alterations in an individual subset of tumor-associated cells [29]. Collectively, these findings show that COX-2 and immune cells share common signaling pathways in mammary carcinogenesis, which are associated with changes in immune cell profiles and functionality and the role of COX-2 may allow neoplastic cells to evade attack by the immune system.

In canine mammary tumors there are only a limited number of studies focusing on the cross talk between COX-2, cancer cells, and immune cells [19, 73] and this topic is incompletely understood. Our team demonstrated a significant association of high COX-2 immunoexpression with CD3^+^ T-lymphocytes [19] and MAC387 macrophages [73]. Tumors with concurrent high COX-2/CD3 and high COX-2/MAC were statistically associated with variables of tumor aggressiveness (high histological grade of malignancy, presence of neoplastic intravascular emboli, and presence of lymph node metastasis) and shorter overall survival of animals [73]. These findings suggest that, similarly to human breast cancer, T-lymphocytes, macrophages, and COX-2 share functions in canine mammary carcinogenesis.

### 4.2. Inflammatory Infiltrates and Receptor Tyrosine Kinases (RTKs)

Receptor tyrosine kinases (RTKs) are cellular proteins that have been intensively studied [52, 74, 75, 123]. Their role in the control of cellular growth and differentiation is central to all organisms and has been found to participate in human and animal neoplastic diseases [124–126].

There are several mechanisms by which tyrosine kinases might acquire transforming functions. RTKs are essential components of cellular signaling pathways that are active during embryonic development and adult homeostasis [127]. Due to their roles as growth factor receptors, many RTKs have been implicated in the onset or progression of various cancers, including mammary cancer, either through receptor gain-of function mutations in the corresponding genes or through receptor/ligand overexpression by autocrine-paracrine growth factor loops [128, 129].

The RTKs family include the human epidermal growth factor receptor (EGFR) family with its members HER-1/EGFR and HER-2, HER-3, and HER-4; platelet-derived growth factor receptors (PDGFR, which include c-kit); fibroblast growth factor receptors (FGFRs); VEGF; hepatocyte growth factor/scatter factor receptor (HGF/SFR); ephrin receptors (Ephs); and the insulin receptor [128, 130, 131].

Here, we will only focus on the most important RTKs in mammary carcinogenesis, the EGFR and c-kit which are often overexpressed or mutated in this type of tumor [52, 126, 132, 133], since VEGF is already described above.

In human breast cancer, RTK signaling has been described as being implicated in differentiation and migration of immune cells into tumor sites contributing to the immune balance from activation to tolerance which is implicated in tumor progression [75, 78, 134, 135]. Consequently, a number of tyrosine kinase inhibitors (TKIs) have been developed which, besides direct anticancer effects, also increase the number and function of effector immune elements, while decreasing the amount and function of suppressor immune cells [81, 136].

Studies in preclinical breast cancer mouse models demonstrated that trastuzumab (monoclonal antibody against HER2) and cetuximab (monoclonal antibody to EGFR/HER-1) both have an important role in tumoral innate and adaptive immunity, inducing natural killer (NK) cells and cytotoxic as well as Th1 T-lymphocytes activity [137–140].

The great homology in the EGFR family among humans and dogs [126] prompted us recently to produce a recombinant canine anti-EGFR antibody for canine mammary tumor treatment [125].

Several lines of evidence suggest that ligands to EGFR family molecules (except HER-2) can be produced by nontumor cell types. The infiltrating T-lymphocytes in human breast cancer can produce EGF [76] and analysis of breast tumor explants revealed that EGF is also produced by TAMs [74, 75, 141]. In fact, the important role of macrophage-secreted EGFR ligands in malignant mammary tumor progression leads to increased carcinoma cell invasion and metastasis [75].

Interestingly, one study showed that even FoxP3^+^ Treg cells express EGFR under inflammatory conditions. The stimulation with the EGF-like growth factor amphiregulin markedly contributes to the enhancement of Treg cell functions* in vitro* and in a tumor vaccination model [77].

In canine mammary cancer there is only one study suggesting that concurrent COX-2/EGFR positive immunoexpression is associated with higher number of intratumoral CD3^+^ T-lymphocytes. In this work, tumoral CD3^+^ T-lymphocytes may be influenced by inappropriate expression of COX-2/EGFR. COX-2 overregulation and the resulting increase in PGE2 levels induced overexpression of EGFR pathways and may represent a strategy adopted by tumors that contributes to the evasion of tumor-specific immune response [19].

In several human tumors, including breast cancer, the stem cell factor (SCF) that triggers the c-kit signaling pathways has been described as possibly being involved in the complex relationship between immune cells and tumor cells in the tumor microenvironment [78, 134]. c-kit upregulation, on DCs, induces the activation of several signaling pathways that block IL-12 and promote IL-6 production. c-kit dependent signaling supports an immune twisting toward Th2 and Th17 subsets and away from Th1 responses. The cytokines produced induce T-cell tolerance and contribute to cancer development [78–80].

Sunitinib and sorafenib, multikinase inhibitors that block, among others, the VEGF and c-kit receptors [81], have the ability to modify the mammary tumor microenvironment in multiple ways, including the alteration of immune cell infiltration by immune subset conditioning [142]. These TKIs may contribute to the enhancement of Th1 and CD8^+^ T-cells intratumoral infiltration with cytolytic activity against the tumor [81, 82]. Sunitinib could also block the conversion of conventional CD4^+^FoxP3^−^ T-cells into CD4^+^FoxP3^+^ Treg cells [81]. Studies* in vitro* and using tumor-bearing mice showed that c-kit inhibitors may induce a reduction of Treg cell number, decreasing consequently the expression of immunosuppressive cytokines (IL-10, TGF*β*) [83]. Furthermore, in mice, sunitinib prompted antiangiogenic effects and promoted direct proapoptotic properties, resulting in a decline of mammary tumor progression. Regarding TAMs, their density was slightly reduced under sunitinib treatment [84].

Canine mammary tumor studies on the interplay between c-kit and tumor immunology are scarce. However, one study demonstrated a positive correlation between CD3^+^ T-lymphocytes and c-kit immunoexpression. Tumors with high CD3/c-kit were associated with tumor aggressiveness (high histological grade of malignancy, presence of neoplastic intravascular emboli, and presence of lymph node metastases) and shorter overall survival of animals. The results of this work are a first attempt to explore the possible common signaling pathways between c-kit and immune system in canine mammary carcinogenesis [52].

Therefore, the findings described above prove that RTK pathways not only are important for the remodeling of mammary tumor microenvironment but also could be a very important target for tumor immunological therapy [135].

## 5. Concluding Remarks

Cancer can take advantage of the stromal inflammation [143]. T-lymphocytes, macrophages, and other inflammatory cells in human and the dog mammary tumor microenvironment acquired protumorigenic properties that are crucial to fuel the major biological processes involved in tumor development, progression, and metastasis [14, 46, 143, 144].

The involvement of inflammatory cells in tumor hallmark acquisitions by supplying proangiogenic growth factors, cytokines, and proteases and the recent success of checkpoint inhibitors in clinical oncology approve the great relevance of the dark side of immune regulation for driving cancer.

The similarities described above between humans and dogs prove the value of dog as an important translational model for comparative oncology in the study of the molecular signaling based on tumor immunosuppression. In the future continuing research on this topic seems to be relevant in order to find novel immunotherapies that may target tumor microenvironment interconnected pathways.

## Figures and Tables

**Table 1:** Relationship between T-lymphocytic and macrophages infiltrate and tissue biomarkers in human and dog mammary tumors.

Tissue biomarkers	T-lymphocytic and macrophages infiltrate	
	Human breast cancer	Canine mammary tumors
Angiogenesis	(i) T-cells can secret VEGF [39] (ii) Treg cells release TGF*β*1 and induce VEGF expression [40] (iii) M2 macrophages are responsible for the production of VEGF, uPA, MMP9, and CCL18 promoting tumor neovascularization [48, 51]	(i) Positive correlation between CD3^+^ T-cells, VEGF, and microvessel density [52] (ii) M2 macrophages infiltration is associated with VEGF expression [56]

Invasion and metastasis	(i) IL-10 produced by Th2-polarized CD4^+^ T-lymphocytes promotes M2-TAMs polarization, enhancing metastasis through EGFR signaling activation [60] (ii) IL-1*β* elicits IL-17 expression from T-cells, resulting in expansion and polarization of neutrophils that have the ability to suppress cytotoxic CD8^+^ T-lymphocytes and favors metastatic spread [61] (iii) MMP-2, MMP-9, and CCL18 produced by macrophages increase tumor cells motility and invasiveness [62, 63]	(i) CD4^+^ T-cells count and TAMs density are higher in metastatic CMT; CD8^+^ T-cells count is higher in tumors without metastatic behavior [64–66]

COX-2	(i) COX-2-derived PGE2 enhances the production of IL-4, IL-5, and IL-10 by Th2 cells and inhibits the antitumor Th1 cytokines (IFN-*γ* and IL-2) [67, 68] (ii) PGE2 inhibits CD8^+^ T-cells proliferation and antitumor activities [69] (iii) COX-2/PGE2 pathways increase tumor Treg cells infiltration, differentiation, and function [70] and can change the tumor-associated macrophages phenotype from M1 to M2 [71, 72]	(i) Tumors with high COX-2/CD3 and high COX-2/MAC are associated with tumor aggressiveness and shorter OS [73]

Receptor tyrosine kinases	(i) T-lymphocytes and TAMs produce EGFR ligands being involved in tumor progression [74–76] (ii) FoxP3^+^ Treg cells express EGFR under inflammatory conditions, which is related to tumor cells invasion and metastasis [77] (iii) c-kit dependent signaling supports an immune twisting toward Th2 and Th17 subsets and the cytokines produced induce T-cell tolerance, contributing to cancer development [78–80] (iv) c-kit inhibitors induce a reduction of TAMs and Treg cell numbers and contribute to the enhancement of Th1 and CD8^+^ T-cells [81–84]	(i) Concurrent COX-2/EGFR expression is associated with higher numbers of tumoral CD3^+^ T-lymphocytes and characteristics of tumor aggressiveness [19] (ii) High CD3/c-kit tumors are associated with variables of tumor aggressiveness and shorter overall survival of animals [52]

## Abbreviations

cAMP: Cyclic adenosine monophosphate
CCL: Chemokine (C-C motif) ligand
CCR: C-C chemokine receptor
COX: Cyclooxygenase
CSF1: Macrophage colony-stimulating factor 1
DCs: Dendritic cells
EGF: Epidermal growth factor
EGFR: Epidermal growth factor receptor (ErbB1 or HER-1)
EMT: Epithelial-to-mesenchymal transition
Ephs: Ephrin receptors
ER: Estrogen receptor
FGF: Fibroblast growth factor
FoxP3: Forkhead box P3
G-CSF: Granulocyte colony-stimulating factor
HER-2: Human epidermal growth factor receptor 2 (ErbB2)
HGF/SFR: Hepatocyte growth factor/scatter factor receptor
IL: Interleukin
IFN-*λ*: Interferon gamma
M-CSF: Macrophage colony-stimulating factor
MDSCs: Myeloid-derived suppressor cells
MIF: Macrophage migration inhibitory factor
MMP: Matrix metalloproteinase
NF*κ*B: Nuclear factor kappa-B
NK: Natural killer
PDGF: Platelet-derived growth factor
PGE2: Prostaglandin E2
RTKs: Receptor tyrosine kinases
SCF: Stem cell factor
STAT3: Signal transducer and activator of transcription 3
TAMs: Tumor-associated macrophages
TCR: T-cell receptor
TGF*β*: Transforming growth factor beta
TGF*β*R: Transforming growth factor beta receptor
TKIs: Tyrosine kinase inhibitors
Treg: Regulatory T-lymphocyte
uPA: Urokinase-type plasminogen activator
VEGF: Vascular endothelial growth factor
VEGFR: Vascular endothelial growth factor receptor.
